# Memory monitoring and memory control in chronic stroke patients Dissociated processes

**DOI:** 10.1590/1980-57642018dn13-010005

**Published:** 2019

**Authors:** Maxciel Zortea, Graciela Inchausti de Jou, Jerusa Fumagalli de Salles

**Affiliations:** 1 PhD, Graduate Program in Medical Sciences, Faculty of Medicine, Universidade Federal do Rio Grande do Sul, RS, Brazil.; 2 PhD, Graduate Program in Psychology, Institute of Psychology, Universidade Federal do Rio Grande do Sul, RS, Brazil.

**Keywords:** metacognition, memory, stroke, cognitive processes, metacognição, memória, acidente vascular cerebral, processos cognitivos

## Abstract

Memory problems are common in stroke patients, although little is known about how accurately chronic stroke patients can monitor and control memory processes.

**Objective:**

The performance of memory and metamemory in stroke patients and healthy controls were investigated, as well as dissociation between performances.

**Methods:**

10 adults with right hemisphere lesion (mean [M] age=53.2 [SD=9.7]), 10 with left hemisphere lesion (M age=60.4 [SD=6.6]) and 20 healthy participants (M age=56.5 [SD=9.3] with no neurological disease, matched for sex, age and years of education participated in a multiple-case design study. Participants completed a metamemory experimental paradigm, as well as immediate and delayed word recall and recognition tasks.

**Results:**

Data indicated that 10 out of the 20 patients presented significantly lower scores compared to controls, two of which had global deficits (functional association). Functional dissociations between memory monitoring (judgments of learning, JOL), control (allocation of study time) and capacity (cued-recall task) among patients were found for eight cases, suggesting these processes are independent.

**Conclusion:**

These findings reveal stroke patients may have specific metamemory impairment and can contribute to the understanding of cognitive models of metamemory processing.

## RESUMO.

Problemas de memória são comuns em pacientes com AVC, embora pouco se saiba sobre a precisão com que pacientes com AVC podem monitorar e controlar processos de memória.

**Objetivo:**

O desempenho da memória e metamemória em pacientes com AVC e controles saudáveis ​​foram investigados, bem como a dissociação entre os desempenhos.

**Métodos:**

10 adultos com lesão do hemisfério direito (média [M] idade=53,2 [DP=9,7]), 10 com lesão no hemisfério esquerdo (M=60,4 [DP=6,6]) e 20 participantes saudáveis ​​(M=56,5 [DP=9,3 sem doença neurológica, pareados por sexo, idade e anos de estudo, participaram de um projeto de múltiplos casos, completaram um paradigma experimental de metamemória, bem como palavras de recordação imediata e demorada.

**Resultados:**

Os dados indicam 10 dos 20 pacientes apresentados. Escores significativamente inferiores aos controles, dois deles com déficits globais (associação funcional), sendo encontradas dissociações funcionais entre monitoramento da memória (julgamentos de aprendizado, JOL), controle (alocação do tempo de estudo) e capacidade (tarefa de recordação) entre pacientes para oito casos, sugerindo que esses processos são independentes.

**Conclusão:**

Esses achados revelam que os pacientes com AVC podem ter comprometimento específico da metamemória, o que contribui para a compreensão dos modelos cognitivos do processamento de metamemória.

Stroke is one of the primary morbidity-mortality factors in the general population,^1^ and memory problems can affect up to 55% of these patients.^2^ Metamemory has been investigated in stroke,^3^ but mainly using questionnaires and therefore lacking performance information. Metamemory refers to the metacognitive processes of monitoring (a subjective evaluation of one’s memory processes) and control (strategic information and actions that promote adaptation of the individual’s memory performance),^4^ as well as the knowledge about one’s memory.^5^ Although the pre-frontal cortex (PFC) has been classically associated with metamemory functions,^6^ other studies suggest the involvement of other regions, such as the anterior cingulate cortex, insular cortex and the hippocampus.^5^^,^ 7 Because some of these regions are related to memory capacity, this raises questions in relation to what extent memory and metamemory are dissociated processes and to what degree stroke patients can present memory monitoring and control deficits.

Previous comparative-group-design studies^8^^,^ 9 have indicated that stroke patients with confirmed lesions may have diminished memory monitoring accuracy, evaluated via judgments of learning (JOLs), which depends on how this is measured (in absolute or in item-to-item relative terms), as well as patient memory capacity. However, the independence of memory and metamemory monitoring are yet to be examined in this population.

Few studies have reported investigations of memory control processes in stroke patients (for self-efficacy training, see Aben et al.^10^) More specifically, when considering classic study-time allocation (STA) strategies for information to be remembered in the future, there is a gap in the literature, although other populations have been studied. For example, Moulin et al. (2011)^11^ studied patients with mild cognitive impairment (MCI) who had lower memory capacity compared to controls. These patients had adequate sensitivity for JOLs, despite having spent twice as much time as controls restudying the items. This suggests a dissociation between memory monitoring and memory control strategies.

It is relevant to consider that stroke can lead to highly heterogeneous cognitive impairments^12^ and group analyses may have certain limitations. In the Cognitive Neuropsychology approach, the case series and multiple-case methodologies^13^ represent alternatives in these situations and allow testing for functional dissociations between cognitive processes. The aim of this study was to investigate patterns of functional dissociations in memory and metamemory sub-processes. It is expected that memory capacity deficits and metamemory impairments, as well as memory monitoring and memory control impairments, might be dissociated in stroke patients.

## METHODS

### Participants

Two groups participated in the study: stroke patients and healthy controls. Inclusion criteria: for both groups, adults aged 30 to 75 years, right-handed and with four or more years of formal education were recruited. Stroke patients were recruited from a neurology service registry of a public hospital and were in the chronic phase (>6 months post-stroke) and had unilateral lesions circumscribed in the telencephalon or diencephalon. Aphasic patients (assessed with the Boston Diagnostic Aphasia Examination - short version)^14^ were not included. A case-control design was used for the control group, which were matched for sex, age and years of education with the clinical cases. Exclusion criteria: participants of both groups were excluded in case of history of alcohol or illicit drug abuse, abnormal (or corrected-to-normal) visual or auditory functions, diagnosis of depression disorder or other psychiatric diagnosis (or being in use of pharmacological treatment) or neurological history. For the stroke patients, neurological and psychiatric problems were accepted only when occurring after the stroke. Controls were also excluded if they exhibited dementia symptoms (assessed with the Mini-Mental State Exam,^15^ adopting cut-off points from^16^).

### Instruments

The Metamemory Experimental Task:^18^ consisted of a computerized paradigm built in E-Prime 2.0 (Psychology Software tools, Sharpsburg PA, US) and administered on a 15.6’ screen laptop, Arial font, size 32, and black and white background. This paradigm assessed memory and metamemory capacity. Participants completed three practice trials with items not presented in the actual test. Independently of their score the task was started. All items were randomly presented. Participants studied a list of 20 word pairs (cue-target) for a future memory test, one at a time, 8 seconds each, 10 with semantic relation (roda [wheel] - pneu [tire]) and 10 without semantic relation (onça [jaguar] - flor [flower]). Norms of Salles et al. (2008)^18^ were used to form the word pairs. After the presentation of all pairs, participants made judgments of learning (JOL) (memory monitoring score) for each item individually. Only the cue was presented (pneu - ?) and they were instructed to answer “how probable is it that you will remember the second word of this pair in a while?” based on a 4-point Likert scale (1 - I am sure I will not remember; 2 - It is unlikely I will remember; 3 - It is quite likely I will remember; 4 - I am sure I will remember). Participants answered orally and the researcher registered the response on a keyboard. After judging all items, a memory cued-recall test was administered for each item individually. Again, participants answered orally and the researcher recorded the response on a sheet of paper, so the participant did not receive feedback. “Don’t know” and guesses were accepted answers. Next, 10 word pairs pre-selected out of the 20 initial items (5 semantically related and 5 without semantic relation) were randomly presented again in a self-paced restudy procedure. Participants viewed one pair at a time and pressed a button to go to the next pair as soon as they considered they would be able to remember the target in a future cued recall re-test. Mean time spent on each pair was considered the restudy time (RST) measure (memory control score). After all pairs were re-studied, a second cued recall test was administered for all 20 initial items. This score was not included in the analyses.

Two measures of metamemory monitoring accuracy were calculated. Relative (REL) accuracy was calculated based on the type-II signal detection theory, using the following formula of the area under the ROC curve:

$$\mathrm{REL}=1/2\Sigma \left[\left(\mathrm{Fi}+1\right)-\mathrm{Fi}\right]\left[\left(\mathrm{Hi}+1\right)\mathrm{Hi}\right]$$

Where F refers to false alarm rate and H to hit rate, while i is a given point on the ROC curve according to the Likert scale points (1, 2, 3 or 4) of the JOL (sensitivity and specificity indices were not calculated due to few points on the curve, although the AUC is a valid measure to detect how accurate each participant judged the probability of successfully remembering the items).^19^ Absolute (ABS) accuracy consisted of the mean proportional difference between the magnitude of the judgments (JOL) and the memory scores (recall) (as in Moulin et al., 2011.^11^ The following formula was applied:

$$\mathrm{ABS}=\left\{1/3\left[\left(1/n\Sigma \mathrm{xi}\right)-1\right]\right\}-\left[1/n\left(\Sigma \mathrm{yi}\right)\right]$$

Where x is the JOL Likert point attributed to a certain item (i), y is the value (0 to 1) indicating whether the item was correctly recalled or recognized, and n the total number of items. RST-JOLs and RST-cued recall were used as measures of strategic use of study time based on monitoring or memory, respectively, and consisted of Pearson correlations between items’ RST and JOL or cued-recall.

The instrument for Neuropsychological Assessment in Adults (Neupsilin-Af)^17^ was used to assess memory capacity using a list of 9 words presented orally for immediate free recall, delayed free recall about 15 to 20 minutes later, and old/new recognition test with 22 single words to judge. Scores consisted of the sum of correctly recalled/recognized items.

Other instruments for sample characterization consisted of a standard questionnaire for sociodemographic and health data, which included a scale (7-point Likert-like items ranging from 0=never to 4=every day of the week) to assess book, magazine and newspaper reading habits and text and message writing habits. Ten points or higher was considered a high frequency of current reading and writing habits. Depressive symptoms were assessed with the Beck Depression Inventory (BDI-II).^20^ One patient had participated in another study previously, and had depression symptoms indexed by the Geriatric Depression Scale (GDS-15).^21^

### Procedure

After signing the informed consent form, participants answered all instruments within two or three sessions of about 2 hours each, individually, at the university or at home (in a quiet environment) in a semi-random order.

### Data analysis

Dissociations between memory and metamemory processes were identified using two criteria: deviant scores and significant difference between scores. Deviant scores or deficits were identified by means of comparing the case scores to the control group (n=20) using a t distribution which considers sample size (for more details, see the Crawford et al. [2009] method).^22^ For the significant difference between scores, the Crawford et al. (2010)^24^ standardized method was used, which considers the correlation between scores. A single dissociation consisted of deficit in one function or at one task and preserved performance in the other, in a single subject; a double dissociation consisted of diametrically opposite performances in two cases: one with preserved performance for function X, but impaired for function Y; and the other impaired for X and preserved for Y. In addition to the case analysis, Spearman’s correlation tests were implemented to investigate the relation between sociodemographic data and memory and metamemory in the clinical group.

### Ethical considerations

This study was approved by the Research Ethics Committee (number 21717) and complied with National Health Council resolutions. All participants volunteered and gave informed consent.

## RESULTS

The final sample was composed of 40 participants: 10 stroke patients with right hemisphere lesion (RHL), 10 with left hemisphere lesion (LHL) and 20 neurologically healthy participants. Table 1 presents data on sample characteristics.

**Table 1:** Sociodemographic and clinical characteristics of patients and control participants (n=40).

	Right hemisphere lesion (n=10)	Left hemisphere lesion (n=10)	Control participants (n=20)	P
Age (years) M (SD)	53.2 (9.7)	60.4 (6.6)	56.5 (9.3)	0.300^a^
Years of education M (SD)	9.3 (3.5)	8.7 (4.4)	9.4 (3.7)	0.847^a^
Sex (M/F)	4/6	4/6	8/12	1.000^b^
Reading habits M (SD)*	5.9 (2.5)	5.8 (4.5)	7.2 (3.2)	0.438^a^
Writing habits M (SD)*	3.2 (2.4)	2.8 (2.5)	4.1 (3.1)	0.498^a^
BDI-II score M (SD)	18.9 (9.3)^1^	19 (8.5)^1^	10.7 (6.5)^2^	0.032^a^

Different numbers indicate significant differences for the post-hoc test Wilcoxon-Mann Whitney test; BDI-II: Beck Depression Inventory;

* Habits during the data collection period;

a Kruskal-Wallis tests;

b Pearson Chi-square test.

Ten patients (half with LHL, Table 1) performed higher than the deficit criteria throughout the experimental paradigm, on all scores, compared to controls. Therefore, they had preserved memory and metamemory abilities. The other 10 patients showed significant deficits compared to controls (p<0.05), except on the following measures: cued recall from the metamemory experimental paradigm and delayed free recall from the Neupsilin-Af. Of the total 10 cases that showed deficits, 2 presented a pattern of association of deficits between processes. Namely, case R8 (“R” indicates right hemisphere lesion) had low relative and absolute accuracy of the JOLs and low performance on the old/new recognition task, which indicates an association between monitoring and memory processes. Another case, L3 (“L” for left hemisphere lesion), had low relative accuracy of the JOLs and low RST-JOL, which indicates an association between monitoring and control processes. Data from these cases are presented in the first part of Table 2.

**Table 2:** Sociodemographic and clinical data of each case according to pattern of deficits in memory control, monitoring and capacity measures (n=20).

Case	Sex	Age	Years of study	RWH score	Months post-stroke	Etiology	Region		BDI-II	Measure with deviant score
**Clinical cases presenting no deficits according to Crawford et al. (2009) criteria (n=10)**										
L1	F	58	5	1	28	H	Subc	Basal ganglia	Minimal	-
L4	F	61	9	9	56	H	Cort/Subc	Parietal	Mild	-
L8	M	52	16	24	21	I	Subc	Internal capsule, globus pallidus and thalamus	Minimal	-
L9	M	60	5	9	36	I	Subc	Corona radiata	Mild	-
L10	M	61	11	11	16	I	Cort/Subc	Insula and internal capsule	Severe	-
R1	F	57	5	4	48	I	Cort/Subc	Temporal	Moderate	-
R2	F	67	11	12	26	I	Cort	Fronto-temporal	Mild	-
R6	F	51	14	12	29	H	Subc	Corona radiata and basal ganglia	Moderate	-
R7	M	38	14	17	35	I/H	Cort/Subc	Fronto-temporo-parietal	Minimal	-
R9	M	60	8	6	24	I	Cort	Parietal	Moderate	-
**Clinical cases that presented deficits according to Crawford et al. (2009)'s criteria and showed functional associations (n=2)**										
L3^a^	F	73	4	4	24	I	Subc	Parieto-occiptal	NA	-
R8^b^	M	57	11	6	11	I	Cort/Subc	Fronto-temporo-parietal	Severe	-
**Clinical cases that presented deficits according to Crawford et al. (2009)'s criteria in only one of the functions evaluated (n=8)**										
Memory monitoring										
R4	F	37	11	11	37	I/H	Cort/Subc	Frontal	Severe	ABS JOL
R5	F	49	7	13	24	I	Subc	Basal ganglia	Mild	ABS JOL
Memory control										
L2	F	70	4	9	7	I	Subc	Thalamus	Severe	RST
L5	F	59	15	12	17	I	Cort	Frontal	Moderate	RST-JOL
L7	M	54	8	0	14	H	Cort/Subc	Basal ganglia and brain parenchyma	Moderate	RST
Memory capacity										
L6	F	56	10	7	14	I	Subc	Internal capsule and corona radiata	Moderate	Old/new recog.
R3	F	61	4	5	22	H	Cort	Frontal	Minimal	Immed. recall
R10	M	55	8	5	23	H	Cort/Subc	Temporal	Minimal	Old/new recog.

RWH: reading and writing habits (total score); BDI-II: classification according to Beck Depression Inventory II; F: female; M: male; I: ischemic stroke; H: haemorrhagic stroke; Cort.: cortical lesion; Subc.: subcortical lesion; NA: participant responded to another depression instrument, the Geriatric Depression Scale (GDS-15) and had a score not suggestive of depression.

a Deficits in memory monitoring and control.

b Deficits in memory monitoring and capacity.

**Figure 1:**
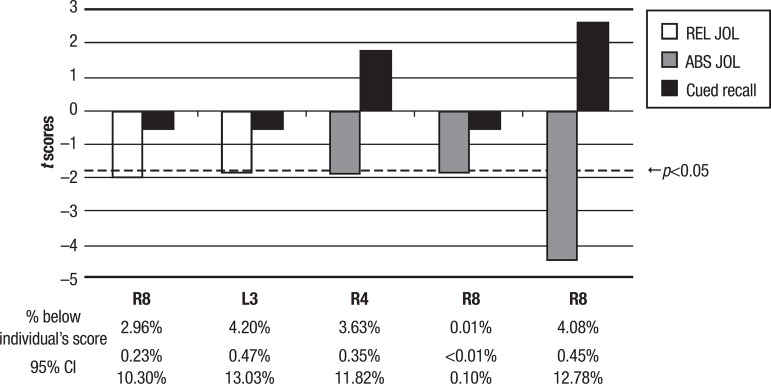
Cases depicting dissociation between memory monitoring and memory capacity. Measures are from the Metamemory Experimental Task. Scores under the dotted line indicate deficit (p<0.05). Percentage of healthy population, estimated from the control group falling below individual’s deviant score and the 95% confidence interval (CI) is displayed at the bottom. REL JOL: Relative accuracy of the JOLs; ABS JOL: absolute accuracy of the JOLs

The remaining eight cases had a profile of functional dissociations. Cases R4 and R5 had low scores for memory monitoring performance, but had scores within the normal range for all other measures. Cases L2, L5 and L7 had low scores for memory control performance, but the scores for the other measures were considered normal in comparison to the control sample. Finally, cases R3, R10 and L6 showed deficit scores in memory capacity performance, but within normal limits for memory monitoring and control measures. This information is given in Table 2. Deficits were found in some measures of memory capacity, monitoring or control, and none of the participants showed profiles of global deficits. Figure 1 depicts cases in which a dissociation (i.e. a significant difference between standardized scores at p<0.05) between memory monitoring and memory capacity measures can be observed. Figure 1 indicates that these cases had low scores for memory monitoring, although memory capacity was spared and significantly higher, relative to the control sample. Among these patients, injuries were predominantly to frontal regions, as well as basal ganglia and temporal-parietal regions (see Tables 1 and 2).

**Figure 2:**
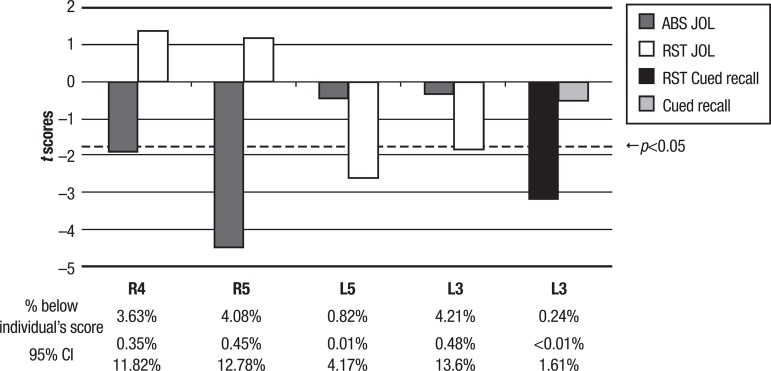
Cases depicting a double dissociation between memory monitoring and memory control (cases R4, R5, L5 and L3) and a single dissociation between memory control and memory capacity (case L3). Measures are from the Metamemory Experimental Task. Score under the dotted line indicates deficit (p<0.05). Percentage of healthy population, estimated from the control group falling below individual’s deviant score and the 95% confidence interval (CI) is displayed at the bottom.

Figure 2 shows a double dissociation between memory monitoring and memory control. The results suggest that cases R4 and R5 used the information from the magnitude of the JOLs to base their restudy time for each item, as expected compared to the control group. However, these patients’ JOLs had very low accuracy in predicting their cued recall capacity. These patients also had lesions to frontal and basal ganglia, respectively. On the other hand, cases L3 and L5 attained good accuracy of their JOLs, but did not base their allocation of restudy time on these judgments. These patients had damage to parietal-occipital and frontal areas, respectively. It is worth noting that cases L3 and L5 were the only ones to show an unexpected positive correlation between restudy time and magnitude of JOLs and cued recall scores. In other words, these patients allocated more time to restudy word pairs they judged easier to recall or that they correctly recalled before. Finally, Figure 2 also illustrates a dissociation between memory control and memory capacity. As can be seen, case L3 demonstrated good capacity for recalling word pairs, but failed to use this information, at an expected level, to guide restudy time.

Lastly, correlations between the dependent variables and age, years of study and reading and writing habits for the clinical sample were analyzed. These date are presented in Table 3.

**Table 3:** Spearman Correlations between measures of memory and metamemory and sociodemographic, health and reading and writing habits data for the clinical group (n=20).

	JOL	FOK	REL JOL	REL FOK	ABS JOL	ABS FOK	RST	RST JOL	RST Cued recall	4-altern. recog	Cued recall
Age	-0.157	0.107	-0.441	0.207	-0.306	0.018	0.231	0.540*	0.283	0.146	-0.406
Years of formal education	0.140	0.106	0.483*	-0.469*	0.022	0.020	-0.467*	-0.066	-0.011	0.285	0.096
Reading habits (before)	0.054	-0.279	0.312	-0.503*	0.006	-0.309	-0.314	0.028	0.249	0.226	0.054
Writing habits (before)	-0.066	-0.206	0.325	-0.092	-0.021	-0.197	-0.030	0.028	0.028	0.076	-0.136
Reading and writing habits (before)	0.017	-0.280	0.331	-0.415	0.022	-0.285	-0.207	-0.013	0.172	0.165	-0.006
Reading habits (after)	0.413	-0.069	0.309	-0.605**	0.276	-0.197	-0.654**	-0.268	-0.092	0.298	0.460*
Writing habits (after)	0.176	0.000	0.456	-0.491*	0.139	-0.058	-0.519*	-0.110	0.045	0.361	0.209
Reading and writing habits (after)	0.255	-0.022	0.486*	-0.599**	0.180	-0.110	-0.598**	-0.184	-0.021	0.351	0.296
Months post-stroke time	0.365	0.000	-0.069	-0.269	0.529*	0.101	-0.276	-0.293	-0.221	0.087	0.333

JOL: magnitude of delayed JOLs; FOK: magnitude of judgments of feeling-of-knowing; REL: relative accuracy; ABS: absolute accuracy; RST: restudy time; RST JOL: correlation between restudy time and magnitude of delayed JOLs; RST cued recall: correlation between restudy time and cued recall scores.

* *p*<0.05;

** *p*<0.01.

Although group analyses are not the focus of this study, performance data for LHL, RHL and controls are presented in Table 4.

**Table 4:** Comparison of performance on memory monitoring, control and capacity scores between groups (n=40).

		RHL (n=10) M (SD)	LHL (n=10) M (SD)	Control (n=20) M (SD)	p
Memory monitoring	JOL	2.14 (0.5)	2.16 (0.8)	2.35 (0.7)	0.530
	REL JOL	0.85 (0.2)	0.86 (0.2)	0.8 (0.2)	0.216
	ABS JOL	-0.01 (0.1)	0.05 (0.1)	0.08 (0.2)	0.155
Memory control	RST	7.91 (2.8)	9.26 (7.3)	6.93 (3.9)	0.143
	RST JOL	-0.61 (0.3)	-0.22 (0.4)	-0.36 (0.3)	0.062
	RST cued recall	-0.56 (0.3)	-0.36 (0.5)	-0.44 (0.3)	0.340
Memory capacity	Cued recall	0.46 (0.3)	0.32 (0.2)	0.38 (0.2)	0.588
	Immed. free recall	4.30 (1.4)	4.80 (1.7)	4.10 (1.1)	0.746
	Delayed free recall	2.20 (1.9)	1.70 (2.0)	1.55 (1.8)	0.286
	Old/new recogn.	14.70 (4.1)	14.40 (2.6)	14.50 (2.4)	0.448

JOL: magnitude of delayed JOLs; REL: relative accuracy; ABS: absolute accuracy; RST: restudy time; RST JOL: correlation between restudy time and magnitude of delayed JOLs; RST cued recall: correlation between restudy time and cued recall scores. p values are based on Kruskal-Wallis tests.

## DISCUSSION

This study demonstrates cases with important impairments in measures of relative and absolute accuracy of JOLs (memory monitoring) and restudy time based on JOLs or cued recall (memory control), which suggest stroke may affect patient metamemory abilities. Moreover, these abilities can be selectively impaired, as revealed by functional dissociations. Multiple-case studies, although sparse in the metamemory literature, are frequently used in Cognitive Neuropsychology approaches aiming to understand cognitive processes and functions. Traditional functional dissociations^23^ were observed between memory capacity, monitoring and control using the same experimental paradigm. These findings corroborate previous studies^8^^,^ 25 and represent an advance with respect to using a different study design and incorporating a measure of memory control. The present research found dissociations between memory capacity and memory control, indicating that although a patient may have normal memory capacity (cued recall), they may not use feedback from their own performance to guide study strategies for retest (RST-cued recall). In the present study, this scenario was found for patient L3. She showed memory monitoring and control deficits and preserved memory performance. It is plausible that her age (73 years) might have been associated with this disruption. According to Castel et al. (2012),^26^ in some cases, social stereotypes and anxiety in relation to memory performance can produce inaccurate memory beliefs and difficulties using strategies appropriately.

In addition, there was strong evidence of a double dissociation between monitoring and control processes. Two patients with RHL based their restudy control strategy on perceived item difficulty, which is considered predictive of adequate memory performance,^4^ although their general perceived item difficulty (through JOLs) was not predictive of memory performance. It should be stressed that both cases showed problems with absolute accuracy, underestimating their recall capacity, which should result, as the model suggests,^4^ in an increase in restudy time. However, this was not the case. On the other hand, two patients with LHL made JOLs with high accuracy of prediction of memory capacity, but failed to control their study time based on this information. The double dissociation found suggests the hypothesis of hemisphere lateralization for memory monitoring and memory control might be correct and, therefore, warrants further investigation.

Sociodemographic and health aspects were expected to have a differential role in explaining deficits and dissociations, but the results were mixed. In Table 1, four out of ten cases showed moderate-to-severe depressive symptoms, although they did not present deficits. Conversely, five out of eight patients that presented some sort of deficit had minimal-to-mild depressive symptoms. There is also no evident pattern for the role of age, years of education or reading and writing habits in the multiple-case analysis. Nevertheless, when considering correlational analyses, age, years of education and reading and writing habits correlated significantly with restudy time, accuracy of the JOL, and cued recall measures (Table 3), revealing its relationship with memory and metamemory scores. Notably, the inverse correlation with restudy time possibly indicates a facilitator effect for encoding verbal information and recruiting cognitive strategies for patients that were involved in intellectual activities. Moderate non-significant correlations were found between depressive symptoms and memory control measures (RST JOL and RST cued recall). These correlations indicate that the greater the depressive symptoms the less patients relied on their judgments or previous memory performance to restudy the word-pairs. This could indicate a specific role of depression in cue-utilization control processes, although this result should be considered cautiously. Further examinations involving larger samples could use age groups (adults and older adults) or educational levels to test in which situations functional dissociations are present and their frequency.

Moreover, our findings suggest patients with brain lesions outside the pre-frontal cortex (PFC), such as exclusive subcortical basal ganglia lesions, had deficits relative to controls for the accuracy of metamemory judgments. As discussed previously, other studies have posited the relevance of other areas for memory monitoring processing, such as the temporal lobe.^27^ Focal lesions in subcortical areas have not often been reported, although Pannu and Kaszniak^6^ addressed disrupted awareness of cognitive deficits in subcortical dementia, HIV and other clinical conditions.

It is necessary to highlight that control measures obtained here are based solely on response time, which is generally slower in elderly people. In addition, it should be considered that the measures of memory monitoring, control and capacity assessed the functioning of specific neural networks, in the sense that we used specific stimuli (verbal input), worked with time-specific information (predictions of future memory) and required particular control behavior (response time). Furthermore, future studies could focus on groups with more restricted lesions, as Modirrousta and Fellows (2008)^8^ attempted in terms of frontal cortex damage. Finally, although depressive symptoms were greater in stroke patients compared to controls, depression is a common comorbidity in chronic stroke patients.^28^

In summary, this investigation represents an advance in terms of employing a neuropsychological case study approach to understand metamemory function using a process-driven experimental task. Moreover, the study delves deeper into the cognitive profile of stroke patients, exposing other areas of possible impaired performance that are mostly understated in neuropsychological evaluation.
